# The Risk of Sexually Transmitted Infection and Its Influence on Condom Use among Pregnant Women in the Kintampo North Municipality of Ghana

**DOI:** 10.1155/2017/8642685

**Published:** 2017-01-26

**Authors:** Martha Ali Abdulai, Frank Baiden, Samuel Afari-Asiedu, Lawrence Gyabaa-Febir, Kwame Kesse Adjei, Emmanuel Mahama, Charlotte Tawiah-Agyemang, Sam K. Newton, Kwaku Poku Asante, Seth Owusu-Agyei

**Affiliations:** ^1^Kintampo Health Research Centre, P.O. Box 200, Kintampo, Brong Ahafo, Ghana; ^2^School of Public Health, Kwame Nkrumah University of Science and Technology, Kumasi, Ashanti Region, Ghana; ^3^Ensign College of Public Health, Kpong, Eastern Region, Ghana

## Abstract

Sexually transmitted infection (STI) affects the reproductive health of both men and women worldwide. Condoms are important part of the available preventive strategies for STI control. The lack of proper risk-perception continues to impede women's ability to negotiate condom use with their partners. This paper is the outcome of secondary analysis of data collected in a cross-sectional survey that explored the perception of risk of STI and its influence on condom use among 504 pregnant women attending antenatal clinic at two health facilities in the Kintampo North Municipality. Consecutively, three Focus Group Discussions were conducted among 22 pregnant women which was analyzed using thematic analysis technique. Multivariate logistic regression analysis was used to identify possible predictors of condom use and risk of STI. Respondents mean age was 26.0 ± 5.9 years. 47% of respondents self-identified themselves as high risk for contracting STI, 50% of whom were married. High risk status (OR = 2.1, 95% CI: 1.1–4.4), ability to ask for condoms during sex (OR = 0.3, 95% CI: 0.1–0.73), and partner's approval of condom use (OR = 0.2, 95% CI: 0.01–0.05) were independent predictors of condom use. Condom use (OR 2.9 (1.5–5.7); *p* = 0.001) and marital status (engaged, OR 2.6 (1.5–4.5); *p* = 0.001) were independent predictors of risk of STI. Women who self-identified themselves as high risk for STI successfully negotiated condom use with their partners. This is however influenced by partner's approval and ability to convince partner to use condoms. Self-assessment of STI risk by women and the cooperation of male partners remain critical.

## 1. Introduction

Globally, over one million people get infected with sexually transmitted infections (STIs) every day [1]. It is estimated that about 357 million people are newly infected with one of four sexually transmitted diseases annually: chlamydia, gonorrhea, syphilis, and trichomoniasis [1]. An estimated 900 thousand pregnant women who were infected with syphilis accounted for about 350 thousand adverse birth outcomes including still births in 2013 [1]. STIs have been documented to have dire consequences on the reproductive health of both men and women, for example, infertility and ectopic pregnancy. They are mainly transmitted through sexual contacts: vaginal, anal, or oral sex [1]. Annually, an estimated 340 million new cases of curable STI are recorded among adults aged 15–49 years worldwide [2]. According to the World Bank, STI, excluding HIV, accounts for 17% of the burden of disease in Africa [3]. It is the second most common cause of healthy life years lost by women aged 15–44 years in Africa, and as such it remains a principal part of the WHO's Global Strategy on Reproductive Health [4]. STI has significantly contributed to the high incidence and prevalence of HIV in Africa [5]. UNAIDS estimates that, in 2015, 36.7 million people were living with HIV, over over 70% of whom were in Africa [6]. HIV accounted for estimated 1.5 million AIDS-related deaths and 2.1 million new infections in the same year [7]. This necessitates the need to control STIs. Apart from abstinence, in the absence of an approved vaccine and an efficacious microbicide for HIV, consistent condom use remains a component of the tools available to prevent the spread of STI including HIV in sub-Saharan Africa, notwithstanding its added benefit of protecting against unintended pregnancies [8].

Several individual, social, and cultural factors affect sexual behavior and influence the choice of preventive methods of sexually transmitted infections including HIV [9]. Therefore an alteration in behavior is required to curtail the spread of these infections. Condom use is generally influenced by a complex interplay of these factors. The Health Belief Model (HBM) stipulates that when individuals perceive that they are at risk of an infection they will be driven to take positive actions towards their health [10]. The HBM has been used in several studies to explore different health behaviors among different populations [11, 12]. The HBM model stipulates that when people believe they are vulnerable to an infection (perceived susceptibility) and believe that the infection has grave consequences (perceived severity) they tend to think that taking an action will reduce their vulnerability to the infection or its severity (perceived benefits) and expect the perceived barriers supersede its benefits. This study study recognizes two levels of interactions: the individual and the partner which influences their perception of risk in sexual behavior and the choice of preventive methods for STI including HIV [13, 14].

Condom use is related to high levels of self-efficacy [15], where self-efficacy reflects a person's level of confidence in his or her ability to perceive risk and control the factors around them that expose them to such risks [16]. The lack of proper risk-perception has been documented to prevent “at-risk” women from using condom on a regular basis [17]. Women who have high perception of risk negotiate condom use with their partners. They are also well-placed to protect themselves from STIs, including HIV. A study by Maharaj and Cleland documented that women who considered themselves at risk of HIV due to their husbands promiscuity were four times more likely to use condoms compared to women who did not [18]. Notwithstanding the benefits of the self-efficacy of women in using condoms, studies have found widespread resistance to use condoms in stable, long-term relationships because of the association of asking for condom use with lack of trust and illicit sex and the fear of an untoward response [18, 19]. In a cross-sectional survey among persons living with HIV (PLWHIV) in Ghana, over 50% of the PLWHIV reported having unprotected anal or vaginal sex, an indication of risky sexual behavior [20]. The 2014 Ghana Demographic and Health Survey (GDHS) emphasizes that, in spite of a relatively high level of knowledge about STI particularly HIV/AIDS, condom use remains inconsistent among both women and men [21]. Indeed, maintaining a relationship can often take precedence over health concerns, predominantly among women who are dependent on men for their economic resources. Only 11% of women and 19% of men with multiple sexual partners have in the past 12 months reported using condoms in their last sexual intercourse [21]. This can contribute to the differentials in the HIV prevalence among women (2.8%) and men (1.1%) [21, 22].

Despite major scientific progress made in STI including HIV prevention, condoms remain a critical part in a comprehensive and practical approach to the prevention of STI and unintended pregnancies. Studies on serodiscordant couples show that consistent condom use significantly reduces the risk of HIV transmission [23, 24]. A recent global modelling analysis estimated that condoms have averted about 50 million new HIV infections since the onset of the HIV epidemic and an expected 225 million couple-years of protection from unintended pregnancies by the end of 2015 [25, 26]. There is paucity of information in Ghana on condom use and the risk of contracting STI. The primary objective of this paper is to explore the predictors and perceptions of condom use and the secondary objective explored how the perceptions of risk of STI influences condom use among pregnant women in the Kintampo Municipality who attended ANC clinic at the Kintampo Municipal Hospital and the Glory Prince of Peace Maternity.

## 2. Materials and Methods

### 2.1. Study Design

We conducted secondary analysis of data from a microbicide survey, cross-sectional study on microbicide acceptability among pregnant women attending antenatal clinic at two health facilities in the Kintampo North Municipality and the Glory Prince of Peace Maternity Home. Both quantitative (paper-based questionnaire survey) and qualitative (Focus Group Discussion) approaches were employed in this study.

### 2.2. Study Setting, Population, Sampling Methods, and Sample Size Calculation

The microbicide survey was conducted in the Kintampo North Municipality in the Brong Ahafo Region of Ghana. It covers a land area of 5108 square kilometers, with about 65% of residents living in rural parts where subsistence farming is the main occupation [27]. Data from the 2014 annual report of Kintampo North Municipality suggest that the population of Kintampo is about 104,571, 23.1% of whom are women of child-bearing age [28]. The municipality is diverse in terms of ethnicity with the main ethnic groups being the Bono's and the Mo's. Christians form the majority (75%) of the residents in this area. The maternal mortality ratio in the Kintampo Municipality has been documented as 347/100,000 [29], which is nearly the same as the national figure of 350/100,000 live births [30]. Majority of the respondents (91%) mentioned the chemical/pharmacy shops as the commonest place where they comfortably buy condoms for use.

The respondents were approached in the capital town, Kintampo, which has one district government hospital, three private clinics, and one maternity home as the main health facilities.

The Kintampo Municipal Hospital, a public hospital, and the Glory Prince of Peace Maternity Home, a private maternity home, were purposively selected as they are the two most visited facilities by pregnant women in the Kintampo Municipality.

This paper is the outcome of secondary analysis of data collected in a cross-sectional study that was undertaken with the primary objective of assessing the acceptability of microbicide among pregnant women attending antenatal clinic in the Kintampo North Municipality [31]. The sample size for microbicide acceptability study was 500 pregnant women and it was expected that this sample will afford the estimation of the level of acceptance of microbicide with a margin of error of 4% (at 95% confidence level), assuming that microbicide was acceptable to 50% of respondents. For this paper, the prevalence of condom use was assumed to be 20%. With a power of 80%, a confidence limit of 95%, and a design effect of 2, at least a sample size of 492 pregnant women are needed. Pregnant women who consented were enrolled, as and when they visited the facility between September and November 2010 for antenatal care.

### 2.3. Data Collection Methods

The study methods for the microbicide survey were triangulated for validation [32]. We used both quantitative (surveys) and qualitative (Focus Group Discussion) approaches for collecting data on condom use (both male and female condoms), attitudes towards condom use, and perception of risk. The results of the quantitative and qualitative findings were compared for similarities and points of divergence.

### 2.4. Quantitative Approach

The survey was conducted between September and November 2010, among 504 pregnant women aged 18–40 years who routinely attended antenatal clinic at the Kintampo Municipal Hospital and Glory Prince of Peace Maternity Home. Respondents were approached to take part in the study at a convenient and enclosed place out of the health facility for privacy during the interview. In the survey, a pretested paper-based questionnaire was administered by trained research assistants in either English or a local dialect (mainly Twi or Mo). Each survey was conducted within 30–60 minutes. The questionnaire explored the sociodemographic characteristics of respondents and that of their partners, attitude towards condom use, and partner-related behaviors towards condom use. Majority of the questions were close-ended. A few of the questions, that is, the respondent's age and partner's age, were open-ended questions.

### 2.5. Qualitative Approach

The survey was followed up with Focus Group Discussions that explored themes that emanated from the preliminary findings of the survey and related literature to better understand and explain the survey results. This provided an exclusive opportunity to understand the human motivations behind certain preferences and sexual behaviors. A purposive sample of 22 pregnant women were selected from the antenatal clinic independent of the survey sample to take part in the FGD. Three FGDs were conducted among women aged 18–40 years living within the Kintampo North Municipality. With age as a guide, the discussion groups were classified into three categories; 18–25, 26–32, and 33–40 years with 8–12 persons per discussion group. Separate discussion was held in the different age groups to ensure relative homogeneity which allows for open discussions. The themes that were explored included general knowledge on condom use, reasons for use or nonuse of condoms, and perceived or experienced consequences of asking for condoms. Responses from these broad themes were regrouped into three categories: reluctance to use condoms, mistrust, and perceptions of inferiority. Other themes were education and most important consideration for using condoms. The FGDs were conducted within a period of 1 month (December). FGDs were held in a convenient and enclosed place out of the health facility for privacy during discussions. All discussions were recorded using digital audio recorder after discussants consented to its use. Each FGD session was conducted within 40–60 minutes. The discussions were facilitated by a moderator and a note taker using a discussion guide and later transcribed verbatim by the researchers.

### 2.6. Data Management and Analysis

The secondary analysis below is built on the HBM particularly; it views condom use as a result of two connected factors: individual and partner-related factors. Individual factors are the sociodemographic characteristics of an individual that incline them to use condoms (age, marital status, educational level, religion, and ability to ask for condoms during sexual intercourse). Partner-related factors include demographic characteristics of the partners that influence the use or nonuse of condoms (partner's age, partner's education, and partner's approval of condoms).

Data from the quantitative survey was double-entered into a password-protected database in Microsoft FoxPro version 9.0. These were verified and cleaned using Microsoft Access. Data analysis was carried out using STATA version 12.

Simple proportion was used to describe categorical and numerical data. Odds ratios and Pearson's chi-squared test were used to test for the strength of the association between the explanatory variables (marital status, respondent age, partner's age, partner's approval of condom use, and ability to ask for condom during sexual intercourse) and the outcome variables; condom use (defined as an affirmative response to the use of condoms within the past three years); and risk of contracting STI (operationalized as an affirmative response to the following questions: “has your partner had any occasional partner within the past three years?”; “does your husband/partner have another wife apart from you?”; in the past three years have you had a reason to think your partner is sleeping with another woman?”; “in the past three years has your partner complained of the following: penile discharge, yellowish/green discharge, offensive penile discharge, genital ulcer, genital wart, painful itchy penis?” Women who self-identified themselves as high risk for STI were classified as “(0) high risk” and those who self-identified themselves as low risk for STI were classified as “(1) low risk”). Bivariate and multivariate logistic regression analysis were used to explore the predictors of condom use and risk of contracting STI. The variables were selected into the logistic regression models if *p* value was ≤0.05 for all test and from literature from earlier studies [33, 34]. The odds ratios were adjusted for age, education, marital status, partners' age, and partners' education.

Thematic analysis was used in the analysis of the qualitative data. This method of analysis involves “identifying, analyzing, and reporting themes within data” [35]. Thematic analysis was used due to its flexibility in providing useful and rich detail to qualitative data [36]. All the qualitative data was coded based on the themes that emerged from the survey. All transcripts were imported into Nvivo 8. These transcripts were read and the responses were grouped according to the major themes. In the process of coding, other emergent themes were added to capture responses that do not fall under any of the initial themes. Before coding, 10% of the transcripts (the same transcripts) were coded by two different researchers. In 90% of the time, the coded transcripts from the two researchers were in agreement. Coded responses were exported and thematically analyzed.

### 2.7. Ethical Approval

Ethical approval for the conduct of this study was obtained from the Institutional Ethics Committee of the Kintampo Health Research Centre. Administrative approvals were also obtained from authorities of the two facilities where the survey was conducted. Written informed consent was sought from each respondent; participants were assured of confidentiality of all the information they provided, minimal or no risk. Voluntary participation was emphasized. As such they will not incur any monetary cost during or after the study neither will they be paid for participating in the study.

## 3. Results and Discussions

The results of the survey are presented as proportions, and odds ratios and the results of the FGDs were reported with illustrative quotes. The results of both the survey and FGDs were triangulated.

### 3.1. Characteristics of Survey Respondents

Table 1 displays the demographic characteristics of respondents. A total of 504 women with mean age of the women as 26.0 years (*standard deviation* = 5.9) were interviewed. Most of the women (57.8%) were between ages of 20 and 29 years with the least (14.3%) of the women aged ≤19 years of age. Over fifty percent (52.6%) of the women had only primary or no education. The women were largely Christians (71.5%) who lived on petty trading (40%) as major occupation. Over half (58.5%) of them were married, with most (55%) of them living within Kintampo Township. Overall forty-seven percent (47%) of women were classified as being at a higher risk of contracting STI while 66% of the respondent did not use condoms in the past three years.

Table 2 presents the bivariate and multivariate logistic regression analysis of condom use and other explanatory variables.

In bivariate analysis, high risk status (OR 3.1 (2.11–4.55); *p* = <0.001), age (≤19 yrs: OR 12 (6.1–23.8); *p* = <0.001, 20–29 yrs: OR 3 (1.7–5.08); *p* = 0.001), highest educational level (beyond primary: OR 3.2 (2.2–4.7); *p* = <0.001), partner's age (18–29 yrs: OR 4.2 (2.9–6.3); *p* = <0.001), partner's level of education (beyond primary: OR 2.1 (1.4–3.2); *p* = <0.001), ability to ask for condom before sex (OR 0.1 (0.1–0.16); *p* = <0.001), partner's approval (OR 0.1 (0.01–0.02); *p* = <0.001), and marital status (engaged OR 3.4 (0.8–14.3); *p* = 0.09), cohabitation OR 1.1 (0.26–4.9); *p* = 0.86) were factors that were significantly associated with condom use.

After adjusting for age, education, partner's age, and partner's education, the explanatory variables were included in the multivariate model at a significance level of 0.05.

After adjustment in multivariate model, high risk status, the ability to ask for condoms during sex, and partner's approval were identified as independent predictors of condom use (Table 2). Women who self-identified themselves as high risk for STI were more likely to use condoms than those women who self-identified themselves as low risk for STI (OR 2.2 (1.1–4.4); *p* = 0.033). Those who could not ask for condoms to be used were less likely to use condoms compared to those who were able to ask for condoms during sexual intercourse (OR 0.3 (0.14–0.73); *p* = 0.010). The women whose partners did not approve the use of condoms were less likely to use condoms compared to those whose partners approved the use of condoms (OR 0.2 (0.01–0.05); *p* = <0.001).

### 3.2. Profile of Respondents' Risk of Contracting STI and Condom Use

Table 3 describes the risk profile of the respondents. Seventeen percent (17%) of women reported having partners who had occasional partners within the past three years. Twelve percent (12%) of women had partners who had over the previous three years complained of at least one of the following: penile discharge, yellowish/green discharge, offensive penile discharge, genital ulcer, genital wart, and painful itchy penis. Eleven (11.3%) percent of the women reported that their partner had another wife or partner apart from them. Overall forty-seven percent (47%) of women were classified as women who self-identified themselves as high risk for contracting STI. Of the women who self-identified themselves as high risk for contracting STI, over sixty percent (65.1%) were married. Majority (62.6%) of women who self-identified themselves as high risk for contracting STI indicated that they had ever used condoms in the past three years as compared to 37.4% of women with low risk of contracting STI. Majority (75.3%) of women who self-identified themselves as high risk for contracting STI compared to 24.7% of women at low risk of contracting STI indicated that avoiding both HIV/STI and pregnancy is important consideration for using condoms. Over sixty percent (67.4%) of women who self-identified themselves as high risk for contracting STI compared to 32.6% of women at low risk of contracting STI reported that they could ask their partners to use condoms. As high as 82.8% of women with high risk partners stated that their partners have to approve of condoms before it can be used.

Using the Pearson chi-square test, age (*p* = 0.003), marital status (*p* = <0.001), partners age (*p* = <0.001), most important consideration for using condoms (*p* = <0.001), ability to ask for condoms during sexual intercourse (*p* = 0.003), partners approval of condom use during sexual intercourse (*p* = 0.001), and whether partner has another wife (*p* = <0.001) were significantly associated with risk of contracting STI.

Table 4 shows the bivariate and multivariate logistic regression analysis of risk of STI and other explanatory variables. Age (≤19 yrs: OR = 3.4 95%; CI: 1.8–6.4), marital status (engaged: OR = 3.7 95%; CI: 2.3–5.8, cohabiting: OR = 1.8 95%; CI: 1.1–2.9), partner's age (18–29 yrs: OR = 4.2 95%; CI: 2.9–6.3), condom use (Yes: OR = 2.5 95%; CI: 1.7–3.6), and partner's approval (OR = 1.6 95%; CI: 1.1–2.3) emerged as factors that were significantly associated with risk of contracting STI. After adjusting for age, education, and partner's age, at a significance level of 0.05, the explanatory variables were included in the multivariate model. Condom use and marital status were identified as independent predictors of the risk of contracting STI (Table 4). Women who used condoms were more likely to be at high risk of contracting STI compared to those who did not use condoms (OR 2.9 (1.5–5.7); *p* = 0.001). Those who were engaged were more likely to be at high risk of contracting STI compared to those who were married (engaged OR 2.6 (1.5–4.5); *p* = 0.001).

### 3.3. Reluctance to Use Condom

The discussants indicated that they were reluctant to use condoms because of their partners. Partners' reluctance to use condoms was associated with hindering maximum sexual satisfaction, because they believed death is unavoidable. Some men were unwilling to use condoms under the pretext that they cannot use it since they were born before condoms were designed. The excerpts below corroborate these findings

*Some of the men who don't like the use of condoms say that they were born before condoms came in existence.* (36-year-old pregnant woman)

*Some men think that whatever the cause of death is, it is still death so they don't really care about the use of condoms.* (27-year-old pregnant woman)

*The men don't like it because they want “skin-to-skin” for satisfaction. Some men will tell you that if you use condoms during sexual intercourse it is like having sex with the condom rather than the woman.* (25-year-old pregnant woman)

Interestingly, some women indicated that they did not like condoms and will never use condoms

*There is no way I will ever buy a condom; I will rather take pill rather than use a condom.* (34-year-old pregnant woman)

### 3.4. Mistrust

Some discussants revealed that both men and women engage in risky sexual activities to avoid creating the impression that they do not trust their partners. They indicated that when a woman asks for condom during sex, it festers mistrust and it gives the impression that she may be hiding a disease condition and suspecting promiscuity of their partner.

*Some think you don't trust them or you might think either they have a sickness or you have it and have not told them about it.* (24-year-old pregnant woman)

*Some women say if a man uses a condom with them it means they do not love and trust them.* (27-year-old pregnant woman)

*As for some men they say they trust their wives so whatever happens for example, pregnancies they don't have a problem with it even if the woman is not interested she will just take it like that.* (35-year-old pregnant woman)

*I think most women sometimes feel if they ask for condoms their partners might suspect them for something they might have done.* (38-year-old pregnant woman)

*Others may also think you don't trust the man. For example, if a man has never worn a condom but suddenly his girlfriend tells him to wear one, it means she does not trust the guy or is suspicious of her guy because she thinks her guy might have another girl somewhere.* (33-year-old pregnant woman)

### 3.5. Perception of Inferiority among Women

Some respondents likened a woman asking for condoms before sex to insubordination. They indicated that the decision to use or not to use condoms sits with the man. These were stated as follows.

*I think that once the man says he will sleep with you he will, there is nothing you can do about it. As a Christian who is supposed to submit to your husband you cannot deny him.* (33-year-old pregnant woman)

*I think part of it is fear because sometimes you are scared to ask for condoms because of possible consequences.* (38-year-old pregnant woman)

*Some men will tell you they married you and not the other way round so you (woman) have no right to tell me to use any such thing (condom).* (30-year-old pregnant woman)

### 3.6. Most Important Consideration for Using Condoms

Some discussants indicated that the prevention of pregnancy and disease was important consideration for using condoms. The following quotes support these findings.

*If the man has not married you sometimes in order to prevent unwanted pregnancy, you use condom to protect yourself.* (21-year-old pregnant woman)

*If your husband is a womanizer it's important to protect yourself from disease by using a condom.* (28-year-old pregnant woman)

### 3.7. Condom Use and Education

A few discussants indicated that educated people are likely to use condoms compared to uneducated people. This is stated as follows.

*If you see a man who likes it [condoms] then he is a bit enlightened and knows how things are moving in the world but for the uneducated ones they will say ‘I will not wear this thing over my penis?', he will never agree.* (39-year-old pregnant woman)

## 4. Discussion

This paper presents results from a secondary analysis of data collected previously in a cross-sectional survey on microbicide acceptability among pregnant women. Overall 66% of the respondents have not used condoms in the past three years. According to the respondents of the FGD, their sexual partners did not like condoms because it reduces sexual pleasure. Interestingly, some women indicated that, beyond their partners not liking condoms, they equally disliked the use of condoms during sexual intercourse. This is not encouraging, given that most of the respondents were young (20–29 years) and were likely to involve in risky sexual behaviors, hence an increased risk of STI including HIV. This finding compares with other studies that have identified reduction of sexual pleasure as a reason for non-condom use among both men and women [37–39]. This finding emphasizes the need to consider barriers to condom use in packaging condom use promotion messages.

The women whose partners did not approve the use of condoms were less likely to use condoms compared to those whose partners approved its use (OR 0.2 (0.01–0.05); *p* = <0.001). The study results show that factors influencing condom use among women revolve around partner-related issues. The qualitative findings underscore the fact that women relegate the decision to use condoms as solely the decision of the man for fear of being tagged obstinate or for the fear of an untoward response. Over the years, women have been the focus of most HIV and STI prevention programs. This finding is important for designing HIV and STI prevention programs. The UNAIDS policy position paper mentions women and girls among the key populations earmarked for HIV prevention [40]. Men have traditionally not been the targets for HIV and STI prevention. However, our study findings highlight the important effect partners exert on decision-making. It emphasizes the critical role men play in the decision to use condoms or otherwise. This therefore requires the involvement of men in designing STI including HIV prevention programs. This finding is corroborated by a study in four countries in sub-Saharan countries that reported that condom use was the individual decision of the male partner [41].

Those who could not insist that condoms are used during sexual intercourse were less likely to use condoms compared to those who were able to ask for condoms during sexual intercourse (OR 0.3 (0.14–0.73); *p* = 0.010). The inability of women to insist on the use of condoms increases their vulnerability to STI including HIV and unintended pregnancies. Gender-based inequalities enshrined in social constructs often lead men to control several facades of decision-making [42]. Undeniably, social constructs that define gender roles influence the degree to which a woman can act independently of a partner's control, influence a partner's actions, and dominate decision-making. Societal constructs may limit women's decision-making power in relationships and increase their risk of HIV and other sexually transmitted infections [43, 44]. The ability of women to ask for condoms during sexual intercourse is dependent on myriad of factors intertwined in religious and customary tenets. Among women, low relationship power has been linked with sexual risk [45, 46]. In the light of our qualitative findings, this study documented similar results with regard to the often cited reasons of not using condoms: promiscuity and mistrust [47]. A woman's ability to negotiate safer sexual practices, particularly condom use, is a vital component of HIV/STI prevention strategies. This finding does not bode well for HIV/STI prevention as well as the prevention of unintended/unwanted pregnancies. It questions the assertiveness of the women to negotiate condom use before sex. Our findings are similar to that of Nakaie et al. in 2014, who conducted a study among women aged 18–49 years to document family planning practices and determine predictors of risk of inconsistent condom use among Cambodian women on ART [48]. It concluded that social, religious, and customary constructs greatly influence various aspects of human behavior including sexual relations. The FGD results highlights that some religious and customary tenets encourage female acquiescence and male superiority. This limits negotiation of condom use among women. For example, by some social, religious, and customary tenets, a good woman is one who is “submissive.” Such women would not deem it right to insist on condom use as this may be likened to challenging the authority of the man. Women admit to trust their partners as such asking for condoms was equated to questioning that trust as highlighted in the qualitative findings [43].

Women who self-identified themselves as high risk for contracting STI were more likely to use condoms than those who self-identified themselves as low risk for contracting STI (OR 2.2 (1.1–4.4); *p* = 0.033). Also, women who used condoms were more likely to be at high risk of contracting STI compared to those who did not use condoms (OR 2.9 (1.5–5.7); *p* = 0.001). These results are encouraging due to the association of risk of contracting STI to condom use. It highlights the ability of women to take action (use condom) owing to a perceived susceptibility to contracting STI including HIV. This finding is corroborated by several studies in South Africa, Zimbabwe, Nigeria, and Ghana [17, 49–52]. Despite the ability to perceive risk, being in stable relationships could present some hindrances to condom use; women who defer the responsibility of using condoms to men as per societal dictates could still be exposed to the twin risk of STI including HIV and unwanted or unintended pregnancy [44]. Our finding emphasizes the need to continually equip both men and women with adequate knowledge on perception of risk in the effort to prevent STIs. Again, it emphasizes the need to build the self-confidence of women to continuously negotiate condom use.

The study findings suggest that women who were engaged to be married were more likely to be at high risk of contracting STI compared to those who were married (engaged, OR 2.6 (1.5–4.5); *p* = 0.001). Women who are engaged to be married have some commitments to their partners but have not completed rites of marriage. When women stay in a relationship for a long time they tend to attain some level of trust as underscored by the qualitative findings. This could increase the risk of these women to contracting STI especially if their partners are not monogamous. According to Gibbs et al., condom use is highly influenced by the length and intensity of relationships: the longer the relationship lasts, the greater the likelihood that condom use will be discontinued [53].

Despite the fact that several sociodemographic characteristics, for example, age and educational status, have been documented to influence condom use [33, 34, 47], our study did not find an association between condom use and these sociodemographic characteristics. This could be as a result of the fact that sociodemographic characteristics work through relationship power to affect condom use. Our finding compares with a study by Pulerwitz et al. who hypothesized that sexual relationship power is crucial to negotiating condom use. Sexual Relationship Power Scale measures power within sexual relationship and asks questions related to relationship control and decision-making dominance. The study found that education and other sociodemographic variables were significantly associated with condom use; however, these associations disappeared when the Sexual Relationship Power Scale was included in the analysis [54].

Several biomedical and behavioral studies have impacted on the course of STI [55–58]. This highlights the need for the combination of biomedical, behavioral prevention packages and structural interventions [59]. Given the critical role condoms play in the comprehensive and practical behavioral approaches to the prevention of STI and unintended pregnancies, our study provides insights into packaging STI and HIV prevention strategies.

The study's findings may have limited generalizability, because this is a cross-sectional study and causality cannot be clearly established. Social desirability could alter the responses given the sensitive nature of the questions; it is conceivable that some respondents may have considered some of the questions asked to be culturally sensitive. These considerations notwithstanding, research officers were trained with skills to build rapport and minimize these effects as much as possible. Despite these limitations, our study provides insight into the factors that can predict condom use and the perception of risk in rural Ghana. It is suggested that interventions that aim to promote risk-perception and safer sexual practices be targeted at both men and women.

## 5. Conclusion and Recommendations

Women who self-identified themselves as high risk for STI were able to successfully negotiate use of condoms by their partners. This is however influenced by partner's approval and ability to convince partner to use condoms. Interventions to educate women on how to conduct a self-assessment of their level of STI/HIV risk could promote the use of condom for prevention. The cooperation of male partners remains critical. Our study implies that STI (including HIV) prevention strategies and interventions that seek to promote condom use should consider the likely influence these factors will have on the woman's assertiveness to use condoms in rural Ghana. It emphasizes that sociodemographic characteristic might not exclusively influence condom use but works through power relationship between partners. It is therefore imperative to consider the identified factors in program development and operation. It emphasizes the need to take advantage of the dual purpose of condom and effectively promote its ability to prevent HIV/STI and as a contraceptive. It underlines the high influence partners/husbands exert on condom use in rural communities in Ghana. Finally it accentuates the importance of building the self-efficacy of women towards condom use.

## Figures and Tables

**Table 1:** Description of participant characteristics.

Characteristics	*n* (%)	Condom use		*p* value
		Yes	No	
*Risk of STI*				
Women who self-identified themselves as high risk for STI (high risk)	239 (47.6)	64 (37.4)	199 (62.1)	<0.001
Women who self-identified themselves as low risk for STI (low risk)	263 (52.4)	107 (62.6)	132 (39.9)	
*Sociodemographic characteristics of respondent*				
Age				
≤19	72 (14.3)	49 (68.1)	23 (31.9)	<0.001
20–29	290 (57.8)	101 (34.7)	189 (65.3)	
30^+^	140 (27.9)	21 (15)	119 (85)	
Highest educational level				
Primary and below	264 (52.6)	58 (22)	206 (78)	<0.001
Beyond primary	238 (47.4)	113 (47.3)	125 (52.7)	
Marital status				
Married	294 (58.5)	64 (21.8)	230 (78.2)	<0.001
Engaged	119 (23.6)	75 (63.0)	44 (37.0)	
Cohabitation	89 (17.9)	32 (36.0)	57 (64.0)	
Religion				
Moslem	141 (28.5)	30 (21.3)	111 (78.7)	<0.001
Christian	354 (71.5)	140 (39.6)	214 (60.4)	
*Sociodemographic characteristics of partner*				
Partner's age				
18–29	179 (35.7)	99 (55.3)	80 (44.7)	0.001
30^+^	322 (64.3)	72 (22.4)	250 (77.6)	
Partners' educational level				
Primary and below	196 (39.0)	47 (24)	149 (76)	0.001
Middle/JSS	122 (24.3)	44 (36.1)	78 (63.9)	
Secondary and above	184 (36.7)	80 (43.5)	104 (56.5)	
Most important consideration for using condoms				
Avoiding HIV/STI alone	215 (43)	39 (18.1)	176 (81.9)	<0.001
Prevention of pregnancy alone	113 (22.6)	48 (42.5)	65 (57.5)	
Avoiding HIV/STI and Prevention of pregnancy	172 (34.4)	84 (48.8)	88 (51.2)	
Ability to ask for condom before sex				
Yes	290 (57.7)	151 (51.9)	139 (48.1)	<0.001
No	212 (42.3)	20 (9.4)	192 (90.6)	
Partner's approval				
No	185 (36.9)	150 (81.1)	35 (18.9)	<0.001
Yes	317 (63.1)	21 (6.6)	296 (93.4)	

Pearson chi-square test was used for these *p* value calculations.

**Table 2:** Bivariate and multivariate analysis of condom use and other variables.

Characteristics	Bivariate OR (95% CI)	*p* value	Multivariate OR (95% CI)	*p* value
*Risk-perception*				
Women who self-identified themselves as high risk for STI (High Risk)	3.1 (2.11–4.55)	**<0.001**	2.2 (1.1–4.4)	**0.033**
Women who self-identified themselves as low risk for STI (Low risk)	1		1	
*Sociodemographic characteristics of respondent*				
Age				
≤19	12 (6.1–23.8)	**<0.001**	9.6 (2.4–39.2)	**0.002**
20–29	3 (1.7–5.08)	**<0.001**	1.8 (0.1–55)	0.18
30^+^	1		1	
Highest educational level				
Beyond primary	3.2 (2.2–4.7)	**<0.001**	1.0 (0.5–2.3)	0.93
Primary and below	1		1	
Marital status				
Married	1		1	
Engaged	3.4 (0.8–14.3)	0.09	8.2 (0.6–114)	0.12
Cohabitation	1.1 (0.26–4.9)	0.9	5.4 (0.4–75)	0.20
Religion				
Christian	2.4 (1.5–3.8)	**<0.001**	1.9 (0.9–4.82)	0.08
Moslem	1		1	
*Sociodemographic characteristics of partner*				
Partner's age				
18–29	4.2 (2.9–6.3)	**<0.001**	1.4 (0.6–3.4)	0.38
30^+^	1		1	
Partner's educational level				
Primary and below	1.7 (1.1–2.9)	**0.021**	1.3 (0.5–3.1)	0.63
Middle/JSS	1		1	
Secondary and above	0.7 (0.5–1.2)	**0.196**	1.6 (0.7–3.6)	0.29
Most important consideration for using condoms				
Avoiding HIV/STI alone	0.2 (0.15–0.37)	**<0.001**	0.5 (0.2–1.1)	0.10
Prevention of pregnancy alone	0.7 (0.48–1.26)	0.3	0.4 (0.2–1.1)	0.08
Avoiding HIV/STI and Prevention of pregnancy	1		1	
Ability to ask for condom before sex				
Yes	1		1	
No	0.1 (0.06–0.16)	**<0.001**	0.3 (0.14–0.73)	**0.010**
Partner's approval				
Yes	1		1	
No	0.1 (0.009–0.02)	**<0.001**	0.2 (0.01–0.05)	**<0.001**

**Table 3:** Chi-square test for risk of contracting STI and other variables.

Characteristics	Risk of contracting STI *n* (%)		*p* value
	Low risk	High risk	
*Sociodemographic characteristics of respondent*			
Age			
≤19	7 (9.7)	65 (90.3)	0.003
20–29	85 (29.1)	207 (70.9)	
30^+^	38 (27.1)	102 (72.9)	
Highest educational level			
Beyond primary	64 (24.2)	200 (75.8)	0.404
Primary and below	66 (27.5)	174 (72.5)	
Marital status			
Married	103 (34.9)	192 (65.1)	<0.001
Engaged	6 (5)	113 (95)	
Cohabitation	21 (23)	69 (76.7)	
*Sociodemographic characteristics of partner*			
Partner's age			
18–29	29 (16.1)	151 (84.9)	<0.001
30^+^	101 (31.1)	222 (68.7)	
Most important consideration for using condoms			
Avoiding HIV/STI alone	77 (35.8)	138 (64.2)	<0.001
Prevention of pregnancy alone	9 (8)	104 (92.8)	
Avoiding HIV/STI and Prevention of pregnancy	43 (24.7)	131 (75.3)	
Ability to ask for condom before sex			
Yes	61 (20.9)	231 (79.1)	0.003
No	69 (32.6)	143 (67.4)	
Partner's approval			
Yes	32 (172)	154 (82.8)	0.001
No	98 (30.8)	220 (69.2)	
Partner ever had occasional partner			
Yes	0 (0)	84 (100)	<0.001
No	263 (62.9)	155 (37.1)	
Partner has another wife			
Yes	0 (0)	57 (100)	<0.001
No	265 (59.3)	182 (40.7)	

Pearson chi-square test was used these *p* value calculations.

**Table 4:** Bivariate and multivariate analysis for the risk of contracting STI and other variables.

Characteristics	Risk of contracting STI *n* (%)		Bivariate 95% CI	*p* value	Multivariate 95% CI	*p* value
	Low risk	High risk				
*Sociodemographic characteristics of respondent*						
Age						
≤19	7 (9.7)	65 (90.3)	3.4 (1.8–6.4)	<0.001	1.2 (0.5–2.8)	0.566
20–29	85 (29.1)	207 (70.9)	1.0 (0.6–1.5)	0.901	0.7 (0.4–1.2)	0.169
30^+^	38 (27.1)	102 (72.9)	1		1	
Highest educational level						
Beyond primary	64 (24.2)	200 (75.8)	1.0 (0.6–1.5)	0.901	1.0 (0.6–1.5)	0.911
Primary and below	66 (27.5)	174 (72.5)	1		1	
Marital status						
Married	103 (34.9)	192 (65.1)	1		1	
Engaged	6 (5)	113 (95)	3.7 (2.3–5.8)	<0.001	2.6 (1.5–4.5)	**0.001**
Cohabitation	21 (23)	69 (76.7)	1.8 (1.1–2.9)	0.012	1.5 (0.9–2.7)	0.138
*Sociodemographic characteristics of partner*						
Partner's age						
18–29	29 (16.1)	151 (84.9)	1.6 (1.1–2.3)	<0.001	0.5 (0.3–1.1)	0.138
30^+^	101 (31.1)	222 (68.7)				
Most important consideration for using condoms						
Avoiding HIV/STI alone	77 (35.8)	138 (64.2)	0.6 (0.4–1.0)	0.081	0.9 (0.6–1.4)	0.571
Prevention of pregnancy alone	9 (8)	104 (92.8)	1.5 (0.9–2.4)	0.084	1.3 (0.8–2.2)	0.317
Avoiding HIV/STI and Prevention of pregnancy	43 (24.7)	131 (75.3)	1		1	
Ability to ask for condom before sex						
Yes	61 (20.9)	231 (79.1)	1		1	
No	69 (32.6)	143 (67.4)	1.2 (0.8–1.7)	0.318	1.3 (0.8–1.9)	0.317
Partner's approval for condom use						
Yes	172 (32.2)	154 (82.8)	1.6 (1.1–2.3)	0.011	0.5 (0.3–1.1)	0.104
No	98 (30.8)	220 (69.2	1		1	
Condom use						
Yes	64 (37.4)	107 (62.6)	2.5 (1.7–3.6)	<0.001	2.9 (1.5–5.7)	**0.001**
No	199 (60.1)	132 (39.9)	1		1	
